# On modeling context-aware social collaboration processes^☆^

**DOI:** 10.1016/j.is.2013.05.007

**Published:** 2014-07

**Authors:** Vitaliy Liptchinsky, Roman Khazankin, Stefan Schulte, Benjamin Satzger, Hong-Linh Truong, Schahram Dustdar

**Affiliations:** Distributed Systems Group, Vienna University of Technology, Austria

**Keywords:** Process modeling, Social context, Collaboration, Visual language

## Abstract

Modeling collaboration processes is a challenging task. Existing modeling approaches are not capable of expressing the unpredictable, non-routine nature of human collaboration, which is influenced by the social context of involved collaborators. We propose a modeling approach which considers collaboration processes as the evolution of a network of collaborative documents along with a social network of collaborators. Our modeling approach, accompanied by a graphical notation and formalization, allows to capture the influence of complex social structures formed by collaborators, and therefore facilitates such activities as the discovery of socially coherent teams, social hubs, or unbiased experts. We demonstrate the applicability and expressiveness of our approach and notation, and discuss their strengths and weaknesses.

## Highlights

• We propose a modeling approach and a visual modeling notation for social collaboration processes.
• We consider collaboration processes as the evolution of a network of documents and people.
• The visual modeling notation is a fusion of statecharts and graph query languages.
• The approach is supported by a formal definition to enable automatic reasoning and verification.
• We present sensitive use cases to demonstrate expressiveness and usefulness of our approach.

## 1 Introduction

Business process modeling (BPM) allows companies to describe and document their enterprise processes. If captured accurately, such knowledge allows to analyze, improve, and execute those processes with higher efficiency. Although a variety of techniques and tools have been introduced for BPM, modeling of highly dynamic non-routine processes, such as human collaboration, is still a subject of discussion in research and very few approaches have been proposed so far [1].

While collaboration in general means working together to achieve a goal [2], [3], with the proliferation of collaboration software, such as groupware or wikis, the manner of human collaboration has taken the form of incremental contributions to a network of shared documents, e.g., source code files, wiki pages and so on. Relations between documents, actors, and other artifacts may influence the collaboration process. For example, some tasks should be done by actors chosen based on social relations, actions on some documents should not be performed before related documents reach certain conditions, or a change in a related document might force to re-do an activity. Moreover, social structures formed by collaborators affect produced network of artifacts. Indeed, Conway’s law suggests that “organizations which design systems are constrained to produce designs which are copies of the communication structures of these organizations” [4]. For example, socially coherent teams tend to produce more seamless solutions. Therefore, a proper modeling of collaboration processes must consider both semantic structures in networks of artifacts and structural formations in social networks formed by collaborators. Although artifact-based process models have already been researched [5], [6], [7], existing modeling approaches do not emphasize the relations between artifacts and actors, and are not capable of capturing complex social structures formed by collaborators.

We thus propose a novel modeling approach and a graphical notation for collaboration processes. The key idea is to treat each document’s evolution as an individual process that is explicitly influenced by the states of related documents and patterns in the surrounding social network. We propose to formalize the relations in line with the data from collaboration software, e.g., two developers can be considered related if they committed code to the same project folder in a source code repository. The amount of such data will grow with social computing pervading the enterprise IT,^1^ thus allowing process modelers to create richer models of people-intensive processes that support information-centric, bottom-up and context-aware and social modeling techniques for collaborative tasks.

The main research contributions of this paper are (i) a novel approach for modeling context-aware social collaboration business processes, (ii) an expressive formalism that allows to define complex dependencies as network of artifacts and people, and (iii) a visual graphical modeling notation. The visual notation is a result of linking two threads of research in a novel way by combining graph query languages and control flow languages. Moreover, with the introduction of the notion of *groups*, this combination is further extended with fundamental concepts of social network analysis by allowing to express such advanced patterns as clique, k-plex, betweenness centrality, closeness centrality, structural equivalence and so on [8]. This paper substantially extends our previous work [9] by (i) introducing the notion of *groups* as first class citizen into the modeling approach, (ii) giving a more detailed discussion of the motivation and related work, and (iii) discussing additional use cases to illustrate the benefits of the concept of groups.

The rest of this paper is organized as follows: Section 2 describes the motivation behind the modeling approach and presents a motivating example. In Section 3 we show the lack of expressiveness in existing modeling approaches with regard to the scenario at hand. Section 4 describes the proposed modeling paradigm and the corresponding graphical notation. Section 5 demonstrates the usability of the approach through realistic use cases. Our modeling approach is critically discussed in Section 6. The paper is concluded in Section 7.

## 2 Motivation

Collaboration is a recursive process composed of human interactions towards realization of shared goals [2], [3]. Groupware and social software foster collaboration of individuals who work across time, space, cultural and organizational boundaries, i.e., virtual teams [10]. Using this type of software, people interact through conversations (e.g., e-mails and instant messages) and transactions (e.g., create/modify/assign/restructure a document) in order to augment a common deliverable, e.g., the documentation of an idea, a technical specification, a source code file, or a wiki page. Typically, such interactions are disorganized, non-routine, and are hard to predict and model. However, as side-effects they produce semantical and social relations between actors and artifacts (e.g., *authorship*, *friendship*). Furthermore, artifacts are usually semantically connected into hierarchical or network structures, e.g., references in wiki pages, or dependencies between software components. Likewise, actors contributing to artifacts form complex social or communication formations, whose structure significantly influences collaboration processes and artifacts themselves. For example, given that a group of collaborators can be represented by a graph with edges denoting regular communication, a group forming a complete graph has more chances to produce a successful artifact(s) than a group forming a sparse graph with many isolates. Patterns of interest differ in artifact and social networks in the sense that structural patterns in artifact networks focus rather on types of relations and artifacts, and their states, while structural patterns in social networks focus on the density of edges by considering single type of relation, e.g., such social formations as clique, k-plex, and notions of structural equivalence, betweenness centrality (broker), and so on [8].

**Fig. 1:**
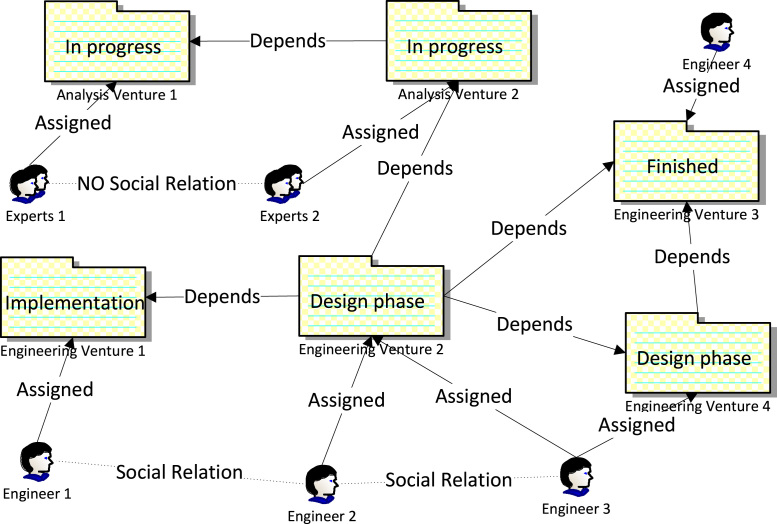
Software engineering collaboration process snapshot.

As a motivating example, let us consider in-house software engineering in a dot-com company. Projects, or ventures, in such a company can be classified as engineering ventures (development of new functionality), or analysis ventures (incident investigation, proof-of-concepts). Both types of ventures produce deliverables, such as source code or technical documentation. Fig. 1 demonstrates a snapshot of a collaboration process as a directed graph of venture deliverables and collaborating actors.

Edges connecting ventures represent functional dependencies (i.e., a venture depends on either an investigation report or a software component produced by other ventures). Edges connecting actors depict social relations, i.e., there is a regular communication over instant messaging channels between them, or they contribute to the same venture. Contrarily, the edge NO Social Relation denotes absence of social ties, e.g., actors never worked on the same venture. Analysis ventures, representing rather creative and non-routine work, can reside only in two possible phases, namely In Progress and Finished, while engineering ventures, representing more structured and long-running work, can reside in more phases, such as Design, Implementation, Testing, and Finished.

Now, let us consider a process modeler that possesses knowledge of the working environment, the culture, and the scale of the company, and aims at modeling the following rules (we refer to them as context dependency rules (CDRs)):

CDR 1. *A venture project team should be notified of any changes in the technical documentation of other ventures it depends on. However*, *if two functionally interdependent ventures share any team members*, *then enforced communication is not required.* This rule ensures proper knowledge sharing between functionally interdependent ventures while avoiding overcommunication. For example, any new technical reports of Analysis Venture 2 should be communicated to the project team of Engineering Venture 2. However, the same synchronization between Engineering Venture 2 and Engineering Venture 4 is not critical, because Engineer 3 is anyway aware of any such changes.
CDR 2. *Venture technical documentation* (*i.e.*, *design*, *or a report*) *should be reviewed by an expert from a functionally dependent venture*, *socially unrelated to the venture team members. Moreover*, *it might be necessary to find a group of such experts.* This rule tries to avoid biased reviews by finding socially unrelated experts. For example, it is more preferable to assign Engineer 4 than Engineer 1, as a reviewer of Engineering Venture 2, as Engineer 4 does not have strong social relations with the Engineering Venture 2 team.
CDR 3. *An engineering venture can be started if at least one venture*, *it depends on*, *has passed* Design phase. This rule defines a balance between total serialization of dependent ventures Design phases, which results in a longer time-to-market, and total parallelization of Design phases, which results in more iterations. For example, Engineering Venture 2 was started upon completion of Design phase of either Engineering Venture 3 or Engineering Venture 1.
CDR 4. Design phase *of a venture cannot be finished if any venture*, *it depends on*, *has not passed* Design phase yet. This rule minimizes chances of potential rework and wasted efforts. For example, Design phase of Engineering Venture 2 can be finished only after Engineering Venture 4 switches to Implementation phase.
CDR 5. *If an engineering venture is in* Implementation phase, *and any of the engineering ventures it depends on has switched back to* Design phase, *then the venture should switch back to* Design phase. This rule covers possible redesign cases and ensures proper handling of late adjustments.
CDR 6. *For each analysis venture it is preferable to assemble a socially coherent team*. Given that a team can be represented as a graph, the extremum of social coherence is a clique, i.e., every two vertices in a graph are connected by an edge. In practice, however, cliques are seldom, and a modeler may want to relax constraints on social coherence by specifying a k-plex, where k-plex can be defined as a group of size *n* having each member connected to at least *n*−*k* other members. Such coherent teams can be selected either for Analysis Venture 1 or Analysis Venture 2. This rule tries to maximize communication within the team and good social atmosphere.
CDR 7. *If a software engineer stops working on an engineering venture during* Implementation phase, *it is necessary to replace her with a structural equivalent*. A structural equivalent is an engineer that has almost the same social neighborhood as the former engineer with respect to dependent project teams. For example, to replace Engineer 2 it is preferable to find an engineer socially connected to both Engineer 1 and Engineer 3. This rule aims at minimizing on-boarding time and restoring communication structures.
CDR 8. *If teams working on two interdependent ventures share no social connections*, *then it is necessary to find a liaison* (*broker*), *who is socially connected to more than 50% of the members of each team*. For example, absence of social ties between groups Experts 1 and Experts 2 may hinder efficient communication thus delaying Analysis Venture 2. This rule tries to cover any structural holes in communication structures formed by collaborating teams.

As it can be seen from the examples above, CDRs allow to capture the knowledge about the impact of social and structural relations on collaboration processes. A formal specification can help to visualize and improve CDRs, thus reflecting management experience in an organization. We argue that a modeling approach, suitable for social collaboration processes, should encompass the following modeling principles that can be abstracted from the examples of CDRs above:

1. *Information-centric*: Collaboration processes should be represented by network of artifacts that originate from and evolve due to collaborative activities, following thus the information-centric perspective. Activity-oriented approaches are difficult to apply to collaboration processes, because it is hard to pre-define exact steps to follow [1]. For instance, people interactions, such as conversations and transactions, in a collaboration process are rather unorganized and unpredictable, therefore, it is easier to capture collaboration artifacts and corresponding social and semantic relations as side effects of interactions. All the exemplified CDRs are based on the information-centric perspective on collaboration processes.
2. *Bottom-up and context-aware*: Modeling an evolvement of a network of artifacts in a holistic view can be a daunting task. Contrarily, neglecting relations completely and modeling the progress of artifacts in isolation leads to context tunneling [11], and therefore ineffective models. A suitable modeling approach, therefore, should model the evolution of each artifact as an individual process explicitly influenced by its neighborhood (i.e., related artifacts), as it is shown in CDR examples 1 and 3–5. This approach allows to describe behavior at the macro-level (network of artifacts) by means of modeling behaviors at the micro-level (evolvement of a single artifact).
3. *Social*: Collaboration processes are influenced by social and communication structures formed by collaborators. Often, advanced non-routine activities are involved, such as discovery of socially coherent teams (CDR example 6) and structural equivalents (CDR example 7), or complex decision-making by exploiting social hubs (CDR example 8), and unbiased experts (CDR example 2). Therefore, the paradigm should promote not only the modeling of a network of evolving artifacts, but also of an evolving network of people. The modeling approach should be able to express not only social relationships between actors involved, but also complex patterns in social networks, such as k-plex, clique, structural equivalence, structural holes and so on.

Moreover, apart from incorporating the mentioned principles, the modeling approach should be backed up by a formal definition to support automatic reasoning, verification, and execution.

## 3 Related work

In this section we discuss the related works with respect to modeling principles outlined in the previous section, and show their shortcomings with regard to their ability to model the exemplified CDRs. To the best of our knowledge, no existing framework is capable of capturing the CDRs defined in this work in a formal and visual manner. In the following, we will discuss activity-oriented business process modeling (Section 3.1), artifact-centric workflows and case handling (Section 3.2), context-aware workflows (Section 3.3), visual graph query languages (Section 3.4), and other approaches which influence our work (Section 3.5). In addition, Table 1 provides an overview of the general ability of the different related works to follow the modeling principles presented in Section 2.

**Table 1:** Overview of covered aspects in the related work.

	Information-centric	Bottom-up & Context-aware	Social
Activity-oriented business process modeling	□	□	□
Artifact-centric workflows			□
Case Handling			□
Context-aware workflows	⊠		□
Visual graph query languages	□	□	⊠
Multiagent systems and speech acts	□	□	⊠

supported, ⊠ partially supported, □ not supported.

### 3.1 Activity-oriented business process modeling

Traditional activity-oriented business process modeling approaches like the Business Process Modeling Notation (BPMN)^2^ allow to model dependencies between processes via messages or events. Asynchronous messaging can be used to partially resemble CDRs, e.g., by sending notifications to related processes. However, it would not provide enough expressiveness and flexibility to capture such rules. Using external events is another way to model such logic, but it would require the specification of events in natural language. Moreover, activity-oriented approaches are difficult to apply for collaboration processes, because it is hard to pre-define exact steps to follow in collaborative workflows [1]. In addition, explicit communication and coordination entities (i.e., events, message channels), intended for publishing information, do not convey any functional load and, therefore, complicate and encumber process models. Agent-based or agent-inspired approaches for coordination of business processes, such as [12], [13], also utilize explicit information publishing entities, thus sharing the same disadvantages.

The Web Services Business Process Execution Language (WS-BPEL or just BPEL) is an executable language standardized by OASIS,^3^ which allows to define business processes based on Web Services. This means that processes in BPEL export and import functionality by using Web Service interfaces exclusively [14]. Major IT companies realized the lack of human interaction support in service-oriented systems and proposed the WS-HumanTask [15] and BPEL4People [16] specifications. While these languages and their extensions (e.g., [17]) allow for interaction with humans in the setting of an SOA, they are not designed to capture CDRs.

### 3.2 Artifact-centric workflows and case handling

Information-centric modeling approaches, such as Artifact-centric workflows [18], [19] and Case Handling [6], can capture the evolution of collaboration entities into formal models in order to provide a higher degree of flexibility than routing-based workflow descriptions are able to provide. Both approaches are examples of entity-centric modeling, which puts entities into the focus of processes and makes use of entity life cycles for dynamic modeling [19].

As the name implies, Artifact-centric workflows are based on (*business*) *artifacts* instead of the task-centric approach usually applied in business process modeling [18]. Artifacts are important (business) objects which have a life cycle and provide information about their relationships to other artifacts as well as in what way and at what time tasks can be invoked on them [20]. Hull [18] makes this more explicit by pointing out that artifact-centric workflows are not merely concerned with modeling process constructs and patterns, but take into account four explicit dimensions: the artifacts themselves, their (macro-level) life cycles, services (tasks) running on artifacts, and associations/constraints.

For this, Artifact-centric workflows capture the relations on a conceptual level using Entity-Relationship models [7], name-value pairs [20], or some Description [18] or First-Order Logics [7]. Life cycles are often depicted using some kind of finite state machines, but other approaches, like the Guard-Stage-Milestone meta-model for life cycles, which is based on Event-Condition-Action (ECA) rules, have also been introduced [18], [21]. Most importantly, the association of services and artifacts can be done either in a *procedural* [20] (also: *imperative*) or *declarative* style [18], [22], [23], [24]. In contrast to the explicit modeling of process constructs as in, e.g., Petri nets, BPEL and BPMN, declarative languages (e.g., [22], [23], [25]) do focus on the goals of the process, i.e., *what* should be done instead of *how* it should be achieved [24]. Example technologies to describe achievable goals using pre- and postconditions are the Ontology Web Language for Web Services (OWL-S) and the Web Service Modeling Ontology (WSMO) [26], [27]. The *ConDec* language [22], which allows both an imperative and declarative modeling of business processes, makes use of Linear Temporal Logic to define declarative process constraints. Using an according model checker, it is possible to verify the correctness of processes and enact it by translating the process into an automaton. Comparable to our work, ConDec defines a graphical notation for such constraints. Due to the nature of the language, this notation is restricted to temporal constraints; social relationships are however not foreseen.

Case Handling distinguishes between the possibility to execute a business process fully automatic (workflow management) and the necessity of human intervention during process runtime (Case Handling) [6], thus allowing a high degree of flexibility and variability. While the former is based on modeled process control structures, in the latter a *knowledge worker* is responsible for actively finding a way to reach the goal of a case. The Case Handling system is a dedicated assistant to the knowledge worker.

(Business) artifacts and cases are based on similar ideas, but cases are more focused on giving the structure and state of a case by data objects and therefore describing these objects in more detail [28]. Data objects are also intended to represent pre- and postconditions. Cases are defined by the activities that need to be executed, data objects, forms which provide activity-based views on data objects, actors, and roles grouping those actors [6], [11]. Associations between single activities are not explicitly modeled, but activities are attached to cases, and data objects are linked to activities. By defining mandatory and restricted data objects, conditions, and precedence relations for single activities, it is possible to model the process underlying a particular case. Conditions are based on data objects’ states and values, and are bound to a particular activity. During design time, roles can be linked to both complex cases and activities; during runtime, concrete actors can be attached to a particular activity. With regard to the work at hand, the evolvement of collaboration entities is captured on a conceptual level using composite cases and ‘is-a’ relationships between roles.

To the best of our knowledge, *condition* elements in neither Artifact-centric workflows nor in Case Handling approaches do allow to specify CDRs. Conditions in Case Handling are defined as sets of bindings where a binding is a set of values for specific data objects. Therefore, it is not possible to define a condition which examines all the objects in a specific relation to the object at hand (CDR example 3), or to specify that *all* the related objects must reside in a specific state (CDR example 4). Conditions in Artifact-centric workflows may be specified in formulas written in First-Order Logic [7]. However, the specification is restricted and does not allow to use quantifiers, which is crucial for expressing CDRs (e.g., CDR examples 3 or 4). Nevertheless, Artifact-centric workflows present an important foundation for our own work, as will be further discussed in Section 4.

### 3.3 Context-aware workflows

Both Artifact-centric workflows and Case Handling are (amongst other reasons) motivated by the assumption that activity-oriented business process models do not capture the workflow context in enough detail and therefore may lead to inefficiencies [6], [18]. Of course, this can be directly traced back to the fact that the modeling perspective in these approaches focuses on other aspects.

According to Dey [29], “context is any information that can be used to characterize the situation of an entity”. Following this definition, workflow context is any information that can be used to characterize the situation of a workflow. Accordingly, Rosemann and Recker define business process context as “The minimum set of variables containing all relevant information that impact the design and execution of a business process” [30].

As a similar notion is also underlying the work at hand, in particular regarding context data related to collaborations, it is worthy to discuss further approaches towards context-aware workflows with regard to the modeling of social collaboration processes. In general, context-aware workflow modeling approaches extend modeling and execution languages like BPMN and BPEL by the means to define context and make use of this knowledge in workflow execution. Very often, this is motivated by some specific domain, e.g., manufacturing [31], [32] or e-health processes [33]. In the following, we will only discuss different approaches in the field which are important to the work at hand; for a thorough discussion we refer to the surveys by Baldauf et al. [34] and Truong and Dustdar [35].

An early, Unified Modeling Language (UML) class diagram-inspired approach to a visual language for context-aware business process modeling has been introduced as part of the *Systemic Enterprise Architecture Methodology* (SEAM) [36]. As it is the goal of SEAM to support human reasoning, a formal reasoning framework is not provided and it is not possible to formally define rules. Instead, some very basic relationships inspired by the means to model composition and dependencies in UML class diagrams are provided. SEAM does not explicitly take care of collaboration between roles, i.e., it is not possible to model CDRs. Instead, different roles are related to each other through actions they are collaborating on.

Saidani and Nurcan [37], [38] regard context-awareness in role-based business process modeling. They allow the definition of location-, time-, resource-, and organization-related context data aiming at the identification of fitting actors for the defined roles. Notably, the authors do allow to state social relationships between actors, but it is not possible to explicitly define CDRs. Comparable to the work at hand, the underlying context model is based on First-Order Logic. However, the authors do not make use of a formal definition of their modeling notation as it is provided in our work. Furthermore, queries are mentioned, but the topic is not discussed on a deep technical level. In general, Saidani and Nurcan do not focus on the actual modeling tasks and therefore do not provide a graphical modeling notation. Furthermore, like in SEAM, they follow a goal-oriented approach while in our work, we focus on information artifacts. Hence, their work should be rather seen as a complementary approach than as a foundation for our modeling notation.

Wieland et al. define context-aware workflows by advocating the augmentation of workflow modeling and execution with information about the physical world [31]. For this, context events, context queries, and context decisions (context-based transition conditions) are added to a workflow model, allowing to define and search context data as well as changes of the control flow. The authors make use of BPMN for process modeling and extend WS-BPEL 2.0 into *Context*4*BPEL*. An XML-based language is used to express context dependencies. Ardissono et al. present the *Context Aware Workflow Execution Environment* (CAWE), which is a complete Service-oriented Architecture extended by capabilities to achieve context awareness [33]. With regard to the work at hand, the context-based adaptation policies are the most interesting aspects of CAWE, as they allow to alter the flow of a workflow execution. These policies are modeled using declarative rules and based on Boolean, context-based preconditions. Abstract activities are used in order to define the generic behavior of a task, thus resembling Artifact-centric workflow modeling languages and Case Handling as discussed above. Furthermore, the authors introduce dedicated models (Role Model, User Model, and Context Model), but do not take into account collaboration issues in them.

In theory, both the work by Wieland et al. and Ardissono et al. could be used as a foundation for drafting and implementing CDRs. However, both frameworks do not explicitly model collaboration dependencies. As a consequence, a resulting model would be somewhat confusingly extensive and therefore hardly intuitive to comprehend. Instead, we decided to draft an independent and therefore lightweight modeling notation as presented in Section 4. The inclusion of explicit information about (social) collaboration allows a much more specific and therefore comprehensible modeling approach.

### 3.4 Visual graph query languages

Conditions in CDRs can be intuitively represented as queries over graph-structured data. Over 25 years, graph query languages have been investigated for expressing graph patterns in various domains such as biological and transportation networks, Semantic Web and many others [39], [40]. In recent years a number of graph query languages have been proposed also for the domain of social networks [41], [42], [43], [44]. Graph query languages do not incorporate any control flow structures, being thus incapable of expressing (business) processes. We review, however, the expressiveness of various graph query languages with respect to complex structural formations.

Many prominent graph query languages, such as **G** [45], GraphLog [46], Lorel [47], StruQL [48], UnQL [49], NAGA [50], Cypher [51] and SPARQL 1.1 [52], are based on *Conjunctive Regular Path Queries* (*CRPQs*), which are in their turn based on *Conjunctive Queries* (*CQ*). A simple example of CQ for finding persons who work on both artifacts Evaluation and Documentation is *x worksOn Evaluation* ∧ *x worksOn Documentation*. CRPQs extend CQs by allowing to query for nodes that are connected by a path satisfying a regular expression rather than relying solely on static paths. Being capable of expressing many graph patterns, CRPQs cannot capture groups of varying size or with inexact topology.

Among non-CRPQ languages greater variability can be observed with respect to graph patterns that can be expressed. For example, GraphQL [53] with repetition of graph motifs allows to define cycles and trees. PQL [54], used for biological networks, can capture along nodes also their neighborhoods of a specified radius. BiQL [42] unifies nodes and edges, which makes it possible to capture even more complex patterns. In QGraph [43], a visual query language employed for social networks data mining tool Proximity [55], graph patterns can have numeric annotations, e.g., “Find all directors that had at least 2 movies each of them winning at least 3 awards”. The Social Networks Query Language (SoQL) [41] is the only graph query language that can describe groups of flexible topology and varying size up to some degree. For example, it is possible to select a clique by specifying a condition on a group as depicted in Listing 1.

Listing 1 Selection of a clique using SoQL.

**Table:** 

SELECT GROUP
FROM GROUP(DISTINCT(X,Y,Z) IN G2)
⋯
WHERE
⋯
ALL SUBGROUPS(U,V) IN G2 SATISFY
(PATH(U TO V AS P1)
COUNT(P1.edges.⁎)<=1)

This query language, however, is not capable to capture more advanced graph patterns, like k-plex. None of the discussed graph query languages has groups as first-class citizens, i.e., it is not possible to specify relations between two or more groups, nor are they designed for CDRs.

### 3.5 Other noteworthy approaches

Several frameworks have proposed for modeling functional or social relations between actors, for example DEMO [56], $I^{⋆}$ [57], EKD-CMM [58], Speech acts [59], [60], or social commitments in multiagent systems [61], [62].

Frameworks modeling functional relations between actors, such as $I^{⋆}$ and EDK-CMM, typically rely on different pre-defined dependency models. $I^{⋆}$ exploits intentional and strategic relationships among actors [57]. It supports a Strategic Dependency model for capturing dependencies among actors for a specific business process design. Dependencies can be due to tasks, resources, goals and soft-goals. Based on that, one can reason how to improve the process/activity. $I^{⋆}$ enables functional relations among actors and it could be used to model the relationships between actors and artifacts but it does not really consider dynamic and social context in our scenario, such as actors are working in the same projects or the evolution of artifacts in connection to other ones. The enterprise model of EKD-CMM supports business processes built atop three main (sub)models: actor/role, role/activity and objects. It focuses on functional relations so static dependencies among actors and artifacts could be modeled. But it does not support social relations and context that links actors and artifacts in particular collaborative tasks. Furthermore, pre-defined actor/role and role/activity models are not well-suited for dynamic relations that we also support in our framework.

Speech acts and social commitments in multiagent systems could be used to model functional relations taken by actors in the form of high-level communication actions. They aim to support both human actors and intelligent software agents so specific languages for human cooperative tasks [59], communicative acts [56], or social commitment protocols [61], [62] have been introduced. But, they are mainly designed for modeling actions, via communication messages, between two individual collaborators. Thus, they do not support well high-level, context-aware interaction patterns among groups like our approach. For example, we could use them to model the request from an actor to another actor, but we could not use them to model the context in which several actors are working on the same document (artifact). Furthermore, they require precise modeling of semantics and action flows to be carried out by collaborators. Due to the inherent ad hoc nature of communication and interactions in collaborations, this would prevent us from modeling dynamic information-centric collaboration actions.

In [63], so-called *batch-tasks* were proposed to allow for a task that is executed for multiple workflow instances at the same time. Other similar approaches can be found in [64]. Some simple CDRs can be covered by batch-tasks, e.g., CDR example 4. For more complex rules, however, this approach is not flexible enough, e.g., because they do not consider artifacts. Team Automata [65], [66] use communication via shared action spaces. Transitions, which include the same external action, are fired simultaneously in these Automata. Alike to batch-tasks, it does not provide the needed flexibility.

While not directly related to our work, the PENELOPE (Process ENtailment from the ELicitation of Obligations and Permissions) language [67] allows to define timing constraints in a manner that could be helpful to define CDR examples 3–5. Interestingly, PENELOPE also allows to automatically generate a state space from the defined timing constraints—this feature has not been foreseen in our work, but would be an interesting aspect for the future work. Finally, the COREPRO modeling framework [68] proposes to model the dependencies between states of related processes via so-called external state transitions. Again, it provides limited expressiveness for describing the dependencies, as it allows to specify only exact external state transitions.

## 4 Modeling paradigm

As discussed in the previous section, related works provide some interesting links and foundations, but none of them provides a holistic approach encompassing all modeling principles and CDR examples discussed in Section 2.

### 4.1 Modeling framework

Our modeling framework is defined as a set of basic modeling elements that a business process modeler can operate with in order to reflect CDRs within business process models:

1. *Collaboration artifacts* and their *states*: Artifacts should represent various aspects and deliverables of collaboration process (e.g., a software component, or a technical design). The states should represent the possible phases of collaborations. *Artifacts* and their *states* may be modeled using existing information-centric approaches, such as Artifact-centric workflows [7], making thus our approach rather complementary than stand-alone. Actors in the modeling framework are modeled as collaboration artifacts as well. This unification simplifies modeling of collaboration processes, as it is easier to predict types (or roles) of involved actors and their states rather than possible actions that comprise collaboration.
2. *Relations*: Relations can be pre-defined (e.g., functional or structural dependency) or dynamic (e.g., temporal or social relations), i.e., produced as side effects of interactions and transactions. Proliferation of groupware and social software boosts the quantity and quality of dynamic relations data, thus empowering process modelers.
3. *Groups*: Groups can be defined as sets of *artifacts* or people shaped by *relations* into formations exhibiting complex structural characteristics.
4. *Context-aware state transitions*: Context-aware state transitions define what *Relations*, *Artifacts* and *Groups* are relevant for a business process at various steps of its execution.

In order to better demonstrate how the framework’s basic modeling elements can be put together to model a business process, we present a graphical notation for the modeling framework. The notation is an extension of the conventional statecharts visual formalism [69]. The choice of statecharts is justified by their information-centric nature and widespread adoption as part of UML.^4^ Being a natural visual representation of the state machine mathematical model, statecharts include the following basic elements: (i) clustered and refined *states*; (ii) state *transitions* composed of *events* (external happenings such as user input or timeout), *conditions* (Boolean expressions over events and state) and *actions* (e.g., sending an e-mail, or assigning a person to a task).

Our graphical notation, dealing with explicit modeling of relations, extends conventional statecharts with a new element Context, graphically depicted as a hexagon. A Context element, being inseparable to a State element, defines relations and artifacts, relevant to a particular state. Each Context element contains a specification of the neighborhood of the artifact (i.e., related artifacts and people) describing the presence of a specific pattern. Essentially, a Context element is a formalization of statecharts’ *conditions*, usually expressed in free-text. Each Context can have several Transition elements attached: If the pattern, described in the context specification, is found in the neighborhood, then all transitions attached to the respective Context element are enabled, otherwise disabled. Similar to State elements in statecharts, Context elements can be clustered using logical AND/OR/XOR operations.

**Fig. 2:**
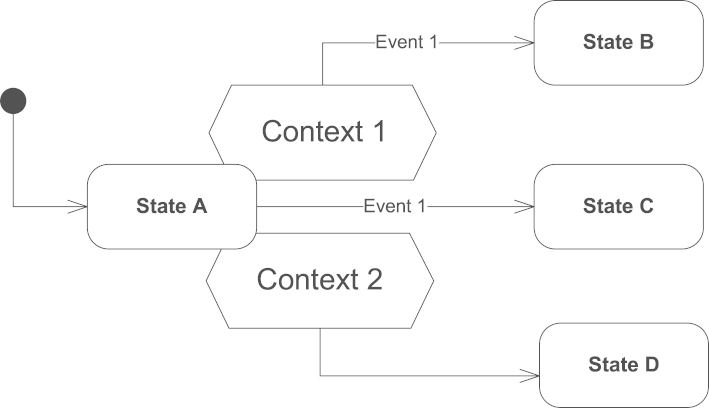
Integration of Context elements into statecharts.

Fig. 2 demonstrates the overall integration of Context element into statecharts (the context specifications are omitted in this figure for the sake of simplicity). Two of the three transitions in the figure are enabled by Context elements. By default, we assume that transitions attached to Context elements have a higher priority over other transitions, but generally it is up to a modeler to define the priorities. Below are enlisted possible transitions in the default prioritization order:

1. If Event 1 is fired and a pattern described in Context 1 is found, then the state machine switches to state B.
2. If Event 1 is fired and a pattern described in Context 1 is not found, then the state machine switches to state C.
3. If a pattern described in Context 2 is found, then the state machine switches to state D. Here we can see that an *event* element is optional, and if absent, then the *transition* is activated at once.

When modeling the behavior of multiple interdependent concurrent process instances, a modeler should assume that state transitions are synchronized, i.e., *every* Context element is evaluated before activation of *any* state transition in any process. Thus, if a process switches to state A and then instantly to some other state, the fact that it has been in state A will be considered.

**Fig. 3:**
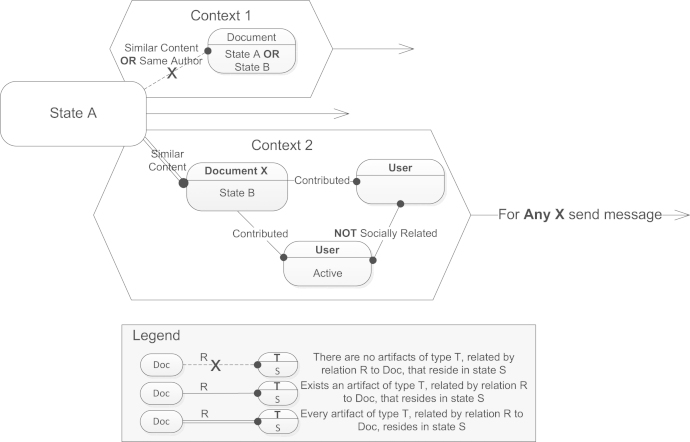
Example of context specifications in Context elements.

We believe that graphs a priori are rather a natural visual medium for describing artifact networks and relations. Therefore, we define a visual graph query language, which is used to define neighborhood specifications in Context elements. A query in the visual language is a directly connected multigraph with labeled edges and nodes. Labels can either denote atomic relations/states/types, or expressions over atomic entities based on propositional calculus expressions. Additionally, labels may be absent in general, denoting a placeholder (e.g., any relation/state/type). An edge direction in a graph is used to depict a non-commutative relation. Query graphs always have one initialized primary element, therefore, graph queries should be interpreted outwards: starting from the central primary element towards most distant nodes. For example, context specifications depicted in Fig. 3 can be interpreted as follows:

• Context 1: If the primary document is in state A, and there are no documents, related by content or author to the primary one, residing either in state A or state B, then the attached transition is enabled.
• Context 2: If the primary document is in state A, and every single document, related by content to the primary one, must reside in state B and have two socially unrelated Authors that contributed to it, one of which is Active, then the attached transition is enabled.

As depicted in Fig. 3, single line edges correspond to existence quantifiers, while double line and crossed dashed edges correspond to universal quantifiers. Nodes in query graphs may be labeled with variables that can later be reused in Conditions and Activities of corresponding Transitions. Since multiple occurrences of a context pattern may be found in the neighborhood, Activities/Conditions may be also extended with quantifiers, i.e., send e-mail to any/every related contributor. Interpretation of the exemplified graph query naturally corresponds to the way we read *First-Order Logic* expressions. *First-Order Logic* with its subsets form a solid foundation for many modeling frameworks and query languages. For our purposes we, however, introduce two extensions that allow for expressing CDRs as introduced in Section 2.

First, we introduce counting and fractional quantifiers that may annotate double line edges. Fractional quantifiers define ratio, while counting quantifiers define exact number of artifacts satisfying given condition. Counting and fractional quantifiers should always be defined with comparison operators {≥,≤,=}, which define, respectively, *at least*, *at most*, and *exactly* conditions. For example, double line edge in Context 2 in Fig. 3 annotated with fractional quantifier ≥70% would be interpreted as “… at least 70% of documents, related by content or author…”.

Universal and existential quantifiers can be considered as special cases of fractional and counting quantifiers respectively, i.e., at least 100% artifacts and at least 1 artifact. Since counting quantifiers are inherently similar to existential quantifiers, one might think of having only one type of edges. We, however, for the sake of simplicity and clarity, consider double line edges to correspond to plural existence of artifacts, while single line edges to singular existence. Similarly, we introduce crossed dashed edges as a special case of double line edges with =0 countable quantifier.

Second, as a means of modeling complex structural formations we introduce the Group element, which defines a set of artifacts or people. Group elements naturally extend graph query notation exemplified so far in the same way as *Monadic Second-Order Logic* (*MSOL*) extends *First-Order Logic. MSOL* allows only existential quantifiers to be applied to set variables. Likewise, groups in our notation can only be defined existentially. Therefore, quantifiers, correspondent to single line, double line and crossed dashed edges adjacent to Group element, are applied to elements of the corresponding group, but not to the group itself. Similarly, state and type labels annotating Group element are also applied to group members (e.g., all group members reside in state A). Double line edges connecting two Group elements may be annotated with two quantifiers as they are adjacent to two plural entities. Along with set variables *MSOL* introduces the atomic formula t∈S, where *t* is a first-order term and *S* is a set variable. Considering practical usefulness of this formula, we introduce an additional edge type, which defines membership. The shape of such edges resembles aggregation association in UML, and the meaning of this type of edges is similar to a weak “has a” relationship.

**Fig. 4:**
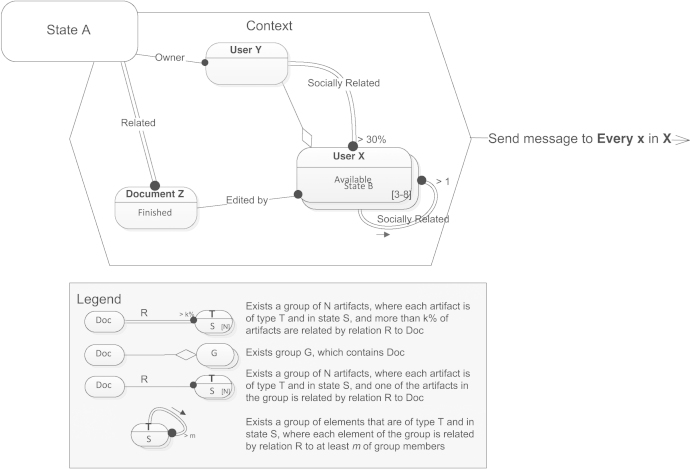
Example of using Group elements.

Let us consider the example depicted in Fig. 4, which shows the selection of a group exhibiting certain structural characteristics. The query described by the example can be interpreted as a conjunction of the following three statements:

• Exists a group X of size 3 to 8 members, such that every member of the group is of type User, and in state Available, and is socially related to at least 2 other group members.
• Every document Z related to the primary one is in state Finished and was edited by a member of the group.
• An owner of the primary document is a member of the group X, and is socially related to at least 30% of the group members.

Interpretation of the exemplified graph query naturally corresponds to the way we read *Monadic Second-Order Logic* expressions. For simplicity, during interpretation it is necessary to define groups before the edges adjacent to them. Quantifiers annotating a loop edge should be interpreted in the order specified by a small arrow attached to the edge.

The success of a modeling approach depends, to a great extent, on the level of simplicity offered. Therefore, we favor simplicity over completeness and impose the following constraints on the queries expressed in the visual language:

• Unlike artifacts, which can be defined with universal quantification by double line edges, groups can be only defined with existential quantification. Being more expressive, universal quantification for groups is rather complex to comprehend.
• Only basic operators from proposition calculus are allowed as literal expressions attached to edges and nodes: conjunction, disjunction and negation. Even though, conditional and biconditional operators may be expressed via the former ones, more complex operators may decrease understanding and make reasoning about the model more difficult.
• Under the Open World Assumption [70], negation may introduce ambiguity, therefore only negation as a failure is allowed, i.e., negation on an edge can be used only if nodes connected by the edge are transitively connected to the central node with non-negative edges.
• During our experiments with the modeling notation we observed that edges with universal (fractional) quantification adjacent to Artifact elements may introduce ambiguity in query graphs with cycles. We can define node level as a length of the shortest path from the node to the primary element. If a double line edge is part of a cycle, then one of its adjacent Artifact nodes should have the lowest level among the nodes in the cycle. In other words, since query graphs are interpreted outwards starting with the primary element, we can say that double line edges adjacent to Artifacts can appear only in those places where they can be interpreted first among edges in the cycle. This rule is not applied to edges with universal quantification adjacent to Group elements, as groups are always defined with existential quantification avoiding thus any ambiguities.

### 4.2 Formal definition

A formal definition of our modeling notation is given below. In order to keep the definition succinct, we omit a formal definition of statecharts, as it is available elsewhere, e.g., in [71]. The formal definition supports automatic reasoning about consistency and correctness of a process at design time, e.g., detection context specifications that can never be reached. Moreover, it enables various optimization techniques, e.g., conversion of a workflow definition to a more compact and simple one.

Definition 1 Labels *L* in a query graph representing relations *R*, types *T* and states *S* of artifacts are defined as

(1) $$\mathrm{Label}\;L\overset{\mathrm{def}}{=}\mathrm{Atomic}\;\mathrm{Condition}|\;\mathrm{Placeholder}|L\;\wedge \;L\;|L\;\vee \;L|\;\neg L,\mathrm{Placeholder}\;\mathrm{denotes}\;\mathrm{any}\;\mathrm{value}\;(\mathrm{nocondition})$$

Definition 2 Edges in a query graph can have one or two attached quantifiers. Beyond standard quantifiers ∀ and ∃ the modeling notation allows generalized quantifiers, similar as proposed in [72]. All extended quantifiers refer to a certain set, which is a whole domain of discourse or a single group in case of Artifact or Group elements respectively. In the following, we define the quantifiers we use by showing the mapping they signify with relation to some arbitrary set *M*:

(2) $$\mathrm{Universal}:\forall_{M}=\{M\}\mathrm{Existential}:\exists_{M}=\{S\subseteq M:S\neq \emptyset \}\mathrm{Counting}:\exists_{M}(⊙n)=\{S\subseteq M:|S|⊙n\},\;\;⊙\in \{>,\geq ,=,\leq ,<\},n\in N\mathrm{Fractional}:\exists_{M}(⊙p\%)=\{S\subseteq M:|S|⊙p|M|/100\},\;\;⊙\in \{>,<\},p\in [0,100]$$

We use capital Greek letters Ξ and Ψ as placeholders for universal, counting, and fractional quantifiers, i.e., Ξ,Ψ∈{∀,∃(⊙n),∃(⊙p%)}.

Definition 3 Edges in a query graph, along with adjacent Artifact elements, are interpreted in First-Order Logic, extended with generalized quantifiers, as follows:

(3) $${\left(a\right)}_{-•}^{R}\left(T,S\right)\overset{\text{def}}{=}\exists x:R\left(a,x\right)\wedge T\left(x\right)\wedge S\left(x\right)$$

(4) $${\left(a\right)}_{=•}^{R}\Xi \left(T,S\right)\overset{\text{def}}{=}\Xi x:R\left(a,x\right)\wedge T\left(x\right)\to S\left(x\right)$$

(5) $${\left(a\right)}_{-\times -•}^{R}\left(T,S\right)\overset{\text{def}}{=}\exists /x:R\left(a,x\right)\wedge T\left(x\right)\wedge S\left(x\right)$$

where, given that graph queries are interpreted outwards from the central primary element (vertex), *a* denotes an already interpreted vertex. Predicates *T* and *S* describe type and state of suitable artifacts respectively. The result of a query graph interpretation is a logical conjunction of the First-Order Logic formulas corresponding to graph edges. Higher priority of edges with universal quantification ensure that the formulas corresponding to these edges always appear at the beginning of the resulting logical conjunction. For double line edges ∀ quantification should be assumed as a default one, i.e., an absence of quantifier annotation is interpreted as ∀ quantifier.

Definition 4 Query graph nodes representing Group elements can be interpreted as follows:

(6) $${\left((T,S)\right)}_{\left[m-n\right]}\overset{\mathrm{def}}{=}\exists G:\forall g(g\in G\to T(g)\wedge S(g))\wedge n<|G|<m$$

[m−n] annotation defines constraints on the group size, where both *n* and *m* can be optional defining thus absence of upper or lower limits. We use capital letters for variables identifying sets (e.g., *G*), and lower case for variables denoting single objects (e.g., *g*).

Definition 5 Edges in a query graph, adjacent to Group elements, are interpreted in Monadic Second-Order Logic, extended with generalized quantifiers, as follows:

(7) $${\left(a\right)}_{-•}^{R}\left(\left(G\right)\right)\overset{\text{def}}{=}\exists g\in G:R\left(a,g\right)$$

(8) $${\left(\left(A\right)\right)}_{-•}^{R}\left(\left(G\right)\right)\overset{\text{def}}{=}\exists g\in G,\exists a\in A:R\left(a,g\right)$$

(9) $${\left(a\right)}_{=•}^{R}\Xi \left(\left(G\right)\right)\overset{\text{def}}{=}\Xi g\in G:R\left(a,g\right)$$

(10) $$\left(\left(A\right)\right)\Psi_{=•}^{R}\Xi \left(\left(G\right)\right)\overset{\text{def}}{=}\Xi g\in G,\Psi a\in A:R\left(a,g\right)$$

(11) $${\left(a\right)}_{-\times -•}^{R}\left(\left(G\right)\right)\overset{\text{def}}{=}R\left(a,g\right)\to g\notin G$$

(12) $${\left(\left(A\right)\right)}_{-\times -•}^{R}\left(\left(G\right)\right)\overset{\text{def}}{=}R\left(a,g\right)\to a\notin A\vee g\notin G$$

Here, similar to previous definition, *a* enclosed into single and *A* enclosed into double round brackets denote an already interpreted artifact or group respectively. Also, ∀ is a default quantifier for double line edges.

Definition 6 In addition to edges defined above, membership and subgroup relations in a graph query can be defined as

(13) $$(a)-⋄((G))\overset{\mathrm{def}}{=}\exists G:a\in G$$

(14) $$((A))-⋄((G))\overset{\mathrm{def}}{=}\exists G:A\subset G$$

Definition 7 Query graph *Q* is a triple defined as follows:

(15) $$\begin{array}{l} {\text{Q}}_{=}^{\text{def}}\left(a,E,V\right),\text{graph}\text{Q}\text{is}\text{Connected},\\ a\text{is}\text{the}\text{pre}-\text{defined}\text{central}\text{primary}\text{vertex}\left(\text{artifact}\right),\\ V\text{is}\text{a}\text{set}\text{of}\text{vertices}\left(T,S\right)\text{and}{\left(\left(T,S\right)\right)}_{\left[m-n\right]},a\notin V,\\ E\text{is}\text{a}\text{set}\text{of}\text{edges}E\subseteq \left\{a\right\}\times \left\{-•,=•,-\times -•\right\}\times \left(V\right)\\ \cup V\times \left\{-•,=•,-\times -•\right\}\times V \end{array}$$

Definition 8 Context element *CTX* in the modeling notation is a composition of query graphs CQ:

(16) $$\mathrm{CQ}\overset{\mathrm{def}}{=}Q|\mathrm{CQ}\prime \;\mathrm{AND}\;\mathrm{CQ}\prime \prime |\;\mathrm{CQ}\prime \;\mathrm{OR}\;\mathrm{CQ}\prime \prime \;|\;\mathrm{CQ}\prime \;\mathrm{XOR}\;\mathrm{CQ}\prime \prime ,\;\mathrm{CQ}\prime =(a\prime ,E\prime ,V\prime ),\;\mathrm{CQ}\prime \prime =(a\prime \prime ,E\prime \prime ,V\prime \prime ),\;a\prime =a\prime \prime ,\;E\prime \cap E\prime \prime =\emptyset ,\;V\prime \cap V\prime \prime =\emptyset$$

Definition 9 Transition element *CT*, attached to Context element *CTX*, can be defined as

(17) $$\mathrm{CT}\overset{\mathrm{def}}{=}(\mathrm{CTX},E,C,\mathrm{AC}),\;E\;\mathrm{is}\;\mathrm{an}\;\mathrm{external}\;\mathrm{event},\;C\;\mathrm{is}\;a\;\mathrm{condition},C:\mathrm{QU}\times \mathrm{ID}\to \{\mathrm{true},\mathrm{false}\},\;\mathrm{AC}\;\mathrm{is}\;\mathrm{an}\;\mathrm{activity},\mathrm{AC}:\mathrm{QU}\times \mathrm{ID}\to \emptyset ,\;\mathrm{ID}\;\mathrm{is}\;a\;\mathrm{set}\;\mathrm{of}\;\mathrm{identifiers}\;\mathrm{attached}\;\mathrm{to}\;\mathrm{vertices}\;\mathrm{in}\;\mathrm{CTX}\;\mathrm{graph},\;\mathrm{QU}\;\mathrm{is}\;a\;\mathrm{set}\;\mathrm{of}\;\mathrm{quantifiers},\mathrm{QU}=\{\mathrm{Any},\mathrm{Every},\mathrm{All}\}$$

In the next section we demonstrate the expressiveness of the defined modeling notation by means of several use cases.

## 5 Use cases

This section describes four collaboration process use cases which demonstrate the application of our modeling approach to various collaboration issues. As it can be seen, the approach allows to easily express the dependency of a process on complex relations in its environment, and to compactly capture the dynamic co-influence between instances of the same process in one model. For clarity, in the use cases we attach to each Context element a free text description of its specification.

### 5.1 Use case—design game

*Goal*: The goal in this use case is to coordinate a design of a complex system consisting of interrelated projects. A set of expert virtual teams thus collaborate to reach a consensus. The assignment relation between teams and projects is one-to-one, but teams can share members. As some projects are dependent, it can happen that changes in the design of one project can be the reason for changes in the design of other ones. Finally, all project designs should be consistent with their dependent ones.

**Fig. 5:**
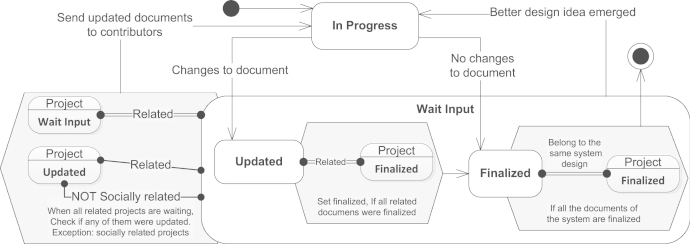
Use case—design game.

*Model*: Each project of this system is regarded as a separate process (see Fig. 5). In the beginning, it is in In Progress state, indicating that the team is currently working on its design. When the team makes some changes to the design and commits it, the process goes into Updated state. If no changes to the design were made, i.e., the existing version was examined and considered valid, then the process switches to Finalized state. The states Updated and Finalized together represent superstate Wait Input, which means that the project design is currently awaiting for some external actions. If the team suddenly decides to update the design (e.g., a better idea emerged), the process goes back into In Progress state.

Now, if the process is in Wait Input state, and if all the related projects are also in Wait input state and at least one is Updated, then the team should check the design of their project against inconsistencies with updated projects. Thus, the updated documents are sent to the team and the state is switched to In Progress. An exception is the case when the project team shares a common expert with the team of an updated project (relation *Socially related*), who is expected to foresee any inconsistencies beforehand. Waiting the related projects to be in Wait Input ensures that all the updates of related documents will be taken into account.

When in Updated state, and if all the related projects are finalized, the process goes into the finalized state, which ensures that if a document spawned no updates among related documents, it will not stay in Updated state.

The system may be considered in the final state when all the projects are in Finalized state.

*Advantages*: This use case demonstrates the modeling of collaboration as ordered iterative communication of project teams towards reaching a consensus. It shows that our modeling approach, as opposed to existing modeling approaches (see Section 3), is capable of expressing universal and existential quantification.

### 5.2 Use case—social selection

*Goal*: The goal of this use case is to support a software development process with the selection of appropriate actors (e.g., developer, adviser, reviewer) based on relations with the other tasks and among the actors. Tasks are related if they belong to the same project, employees are related if they collaborated before.

**Fig. 6:**
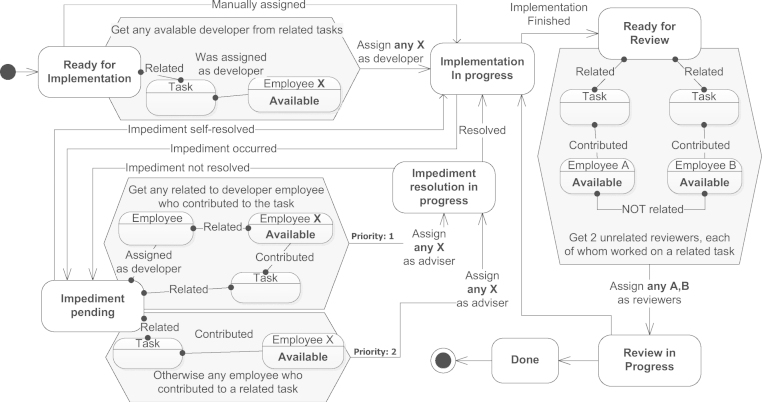
Use case—social selection.

*Model*: Fig. 6 depicts the software development process. At first, the task is in the Ready for Implementation state and is waiting for an appropriate developer to be assigned. Any available developer from a related task is assigned for this role, as he/she is expected to be more productive because of being familiar with some related concepts. Alternatively, a manual assignment is performed. In either case, the process goes to the Implementation in Progress state. An impediment can occur during the implementation (Impediment pending state), in which case an adviser is needed for assistance. An adviser is preferably selected as being related to the developer employee who contributed to a related task, because of joint work experience. Otherwise, any related task contributor is chosen. If the adviser is found, the process goes into Resolution in Progress state, from where it can either go either back to Implementation in Progress or Impediment Pending states, depending on whether the impediment has been resolved. Also, the developer can resolve the impediment by herself if no adviser was found. After the implementation is finished, the reviewers are selected (Ready For Review state): they are desired to have experience with related tasks but be unrelated to each other, which assures unbiased reviews. After the review process (Review In Progress state), either the implementation needs to be revised, or the task is considered finished.

*Advantages*: This use case demonstrates expressiveness of the modeling approach when visualizing a social network environment, allowing thus to model processes that require discovery (e.g., compose a socially coherent team), unbiasedness (e.g., involve independent people), and negotiation (e.g., by exploiting of social hubs). It shows expressiveness of the graphical notation with regards to modeling discovery in a surrounding social network. Contrarily, existing modeling approaches fall short of expressing such patterns in a visual and formal manner (see Section 3).

### 5.3 Use case—dependent components

*Goal*: The goal is to coordinate the development and testing of a software product, which consists of manifold components, some of which depend on others (we assume no cyclic dependencies). The development a component should proceed only when the components it depends on have reached certain progress.

**Fig. 7:**
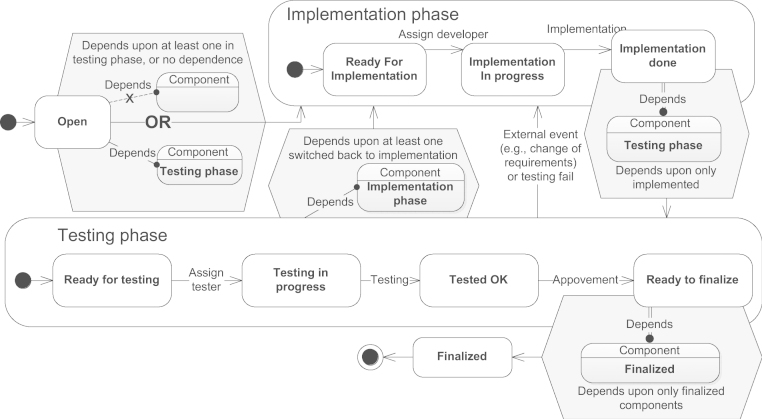
Use case—dependent components.

*Model*: Fig. 7 depicts the process which corresponds to a single component. It starts in Open state and switches over to Implementation Phase in either of the two cases: it does not depend on any components, or at least one component which it depends on is in Testing Phase. This ensures some minimal basis for the development. After Implementation Phase, the component is ready to switch over to Testing Phase, but, first, it should wait for all the components it depends on to be implemented, so the testing covers the combined functionality. The testing phase can reveal some flaws so the component will return into Implementation Phase for fixing those. If, while the component is in Testing Phase, any of the components it depends on suddenly goes into Implementation Phase, then the testing should be stopped in order not to waste the testing effort on outdated components. Lastly, if the component is in Ready to Finalize state, and all the components it depends on are Finalized, then the component can be finalized.

*Advantages*: This use case demonstrates the suitability of the modeling approach for expressing the coordination of project teams towards ensuring consistency and correctness of a complex product. It shows the expressiveness of our modeling notation if comparing it to existing modeling approaches that would capture process coordination either in a text form or via events (see Section 3).

### 5.4 Use case—teams and groups

*Goal*: The goal of this use case is to compose effective teams based on social connections and internal company structure. This use case exemplifies a composition of a development team, a replacement search for a key role (here: SCRUM Master^5^), and the formation of independent expert groups.

**Fig. 8:**
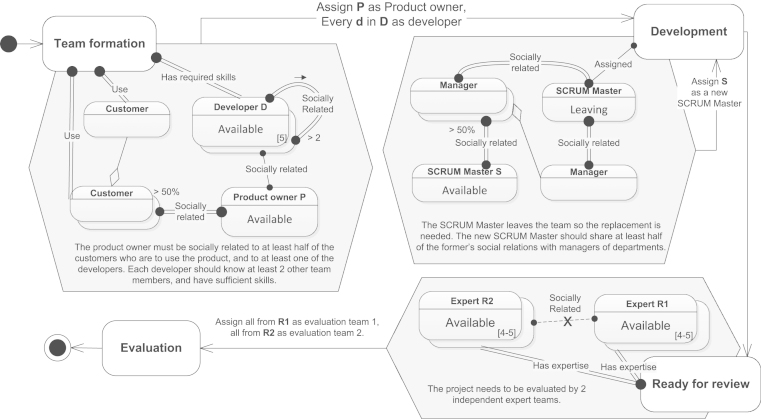
Use case—teams and groups.

*Model*: Fig. 8 shows a simplified software development process with focus on team creation and support. The process starts in Team Formation state where a development team of five people and a product owner are chosen. The product owner should be socially related to at least half of the future users of the product at hand, which ensures more efficient communication of requirements and feedback. The product owner should also know at least one of the developers, so a better contact with the team can be established. Developers in turn should have the skills necessary for the project and should know each other to some extent for easier integration, so the rule states that each developer in a team should be related to at least two others.

To specify the requirement that *all* the customers that will use the product must be included in the group, we need to use an advanced pattern, because having only a double edge from the primary element is insufficient: that would mean that there *exists* a group of customers, all of whom will use a product, but does not imply that all such possible customers will fall into this group. We thus need to use an additional single Customer element defined with a universal quantification, which means that each customer who uses the product is included in the group. The double edge from the primary element should remain to state that the group is restricted only to the customers who will use the product. The similar technique is used in the context with SCRUM Masters.

Once the team is formed, the development phase starts and the process goes into Development state. During this phase it might happen that some member leaves a team for an arbitrary reason, and a replacement has to be found. The use case illustrates such a situation with the team’s SCRUM Master. A criterion for the new SCRUM Master is that she should share at least half of the connections of the leaving SCRUM Master to managers of collaborating departments. This rule aims to retain the pace of issue resolution, should any occur while collaborating with the other teams. After the development phase is over, the process goes into Ready for review state where evaluation teams are composed. Two teams of four to five people should be independent, i.e., a member of one team should not have any connections to the members of the other team, to assure unbiased evaluation. The process can then switch to Evaluation phase and eventually be finished.

*Advantages*: This use case demonstrates the capabilities of the framework to express advanced patterns in social networks, such as 2-plex (development team), broker (product owner), and structural equivalence (new SCRUM Master), as well as conditions involving multiple teams.

## 6 Discussion

Compared with existing approach, the main strengths of our modeling notation are its expressivity and flexibility. First, the modeling notation is capable of capturing complex graph patterns inherent to social networks. Second, the modeling notation goes in line with statecharts by avoiding any domain-specific constructs, making it applicable outside of the social networks domain. Third, it allows to capture the evolution of a network of artifacts, as well as a network of people. Our modeling approach is supported by a formal definition, enabling thus design time reasoning, verification, optimization and efficient execution.

The absence of explicit communication entities (events or messages) in the modeling approach is a strength regarding the clarity of the resulting model, but also a weakness. It allows to provide simple processes coordination and secure encapsulation: a process can modify only its own state, it cannot impact related processes explicitly, similar to Cellular Automata (CA) [73]. However, a modeler cannot immediately see what parts of a business process (states) other processes rely upon. Given that definitions of events and messages represent a process interface, a modeler will not be able to remove or change process states without a certain risk of affecting other models. However, this problem can be remedied with state clustering available in statecharts.

Unlike CRPQ-based languages (see Section 3.4), our visual notation does not have notion of paths defined with regular expressions. Seamless integration of paths requires further investigation with respect to usefulness in the scope of context-aware processes, and is part of our future work. Also, we envision that other additional elements might be added, like aggregation operators to describe the accumulated state of the entire neighborhood. Moreover, among our major interests are the possibility of sharing context elements between parallel processes along with zoom in and zoom out capabilities for *group* elements.

Our modeling framework unifies active and passive entities, i.e., actors and artifacts, and considers them from the perspective of classification (type) and possible states. Correspondingly, our graphical modeling notation employs a single type of shapes for both actors and artifacts in line with statecharts. This approach emphasizes the viewpoint of groupware and collaborative software, where actors are represented simply as user profiles, which are, essentially, also documents. The unification affects slightly the intuitiveness of the modeling notation, as it is not immediately visible which entities are active and which are passive. However, this unification allows for greater flexibility, e.g., it is possible to specify an actor as a central element, modeling thus evolvement of a user profile, or express a semantically coherent group of artifacts. Moreover, it enables a broader use of the framework and potential application to other domains. For example, it is possible to introduce actors that represent software agents, or other types of social entities, such as organizations.

According to the formal definition (see Section 4), our modeling notation incorporates *relation* as a modeling element, but neither types nor semantics of relations are formalized. This makes sociality of the modeling notation somewhat implicit, coming rather from the ability to express common patterns in social networks and their influence on collaboration processes. Being highly dependent on the target domain, semantics of relations between collaborators are left to be defined by the modeler, as well as possible problems to infer those relations. Absence of specific semantics behind relations, again, allows for greater flexibility, enabling a modeler to define and adjust many specific types of relations, such as colleagues, acquaintances, relations denoting mutual dislike or past conflicts and so on. To avoid this, a context taxonomy [37], [74] could be extended to incorporate information about different social relationships.

## 7 Conclusion

This paper proposes a modeling approach and a corresponding graphical notation for creative human collaboration processes. The applicability of the approach was demonstrated through several use cases, and its strengths and weaknesses were discussed.

Comparing to existing approaches, our contribution has two main distinguishable features: it is capable of capturing complex patterns in network of artifacts and people, and it advocates a communication model where a process can modify only its own state and cannot explicitly impact related processes. We have shown that these features are naturally suitable for modeling of social collaboration processes. Although our approach was designed with this focus, we do not exclude its applicability in other areas.

In [75], we first presented an execution framework for our modeling approach in the form of a coordination language. In the future we plan to extend this execution framework with the notion of *groups* in order to allow for coordination of collaboration processes based on complex formations in social surroundings.

## Footnotes

1 http://www.gartner.com/it/page.jsp?id=1470115

2 http://www.bpmn.org/

3 http://www.oasis-open.org/

4 http://www.omg.org/spec/UML/

5 http://scrummethodology.com/scrum-effort-estimation-and-story-points/
